# The impact of a cartoon character on adults perceptions of Children’s breakfast cereals: a randomized experiment

**DOI:** 10.1186/s12937-020-00565-5

**Published:** 2020-05-17

**Authors:** Alejandra Contreras-Manzano, Alejandra Jáuregui, Claudia Nieto, Marissa G. Hall, Jorge Vargas-Meza, James F. Thrasher, Daniel Illescas-Zárate, Simón Barquera, David Hammond

**Affiliations:** 1 Nutrition and Health Research Center, Mexican National Institute of Public Health, Av. Universidad 655 Col, Santa María Ahuacatitlán, 62100 Cuernavaca, Mexico; 2 Department of Health Behavior, Gillings School of Global Public Health, University of North Carolina, Chapel Hill, NC 27599 USA; 3 Population Health Research Center, Mexican National Institute of Public Health, Av. Universidad 655 Col. Santa María Ahuacatitlán, 62100 Cuernavaca, Mexico; 4 Department of Health Promotion, Education & Behavior, Arnold School of Public Health, University of South Carolina, 921 Assembly St, Columbia, SC 29208 USA; 5 School of Demography, ANU College of Arts and Social Sciences, The Australian National University, 9 Fellows Road Acton ACT, Canberra, 260 Australia

**Keywords:** Cartoon characters, Marketing directed to children, Goodness perception

## Abstract

**Background:**

Cartoon characters on processed food packaging increase the perception of product preference among children, but their effect among adults has rarely been examined. We evaluated the effect of a cartoon character on breakfast cereals on beliefs about buying them for children, as well as whether demographic characteristics modified this effect.

**Methods:**

An experimental study was conducted with adults from online consumer panels in Mexico (*n* = 3755). Participants were randomly assigned to a “cartoon” condition (*n* = 1789), in which they viewed a breakfast cereal box with a Minion character on the front of the package, or the “control” condition (*n* = 1966), in which the same cereal box was displayed with no character on the package. Participants were asked: *“Is this a good cereal to buy children?”* with the response options “Yes”, “No”, or “Don’t know”. Multinomial adjusted logistic models regressed responses to this question (Yes = 0, No = 1, 2 = Don’t know) on experimental condition. Differences in the effect of the cartoon character across demographic characteristics were tested by introducing multiplicative interaction terms.

**Results:**

The adjusted model showed that participants in the “cartoon character” condition were 1.67 (1.45–1.94) times more likely to consider the cereal as being “Not good to buy for children” than those in the control condition (*p* < 0.001). This effect was smaller among parents (RRR = 1.39, 1.13–1.72) compared to those without children (RRR = 2.01, 1.63–2.47). No differences were observed in the proportion of participants answering “Don’t know” across experimental groups.

**Conclusion:**

Among this sample of Mexican adults, a cereal with a cartoon character on the packaging was more often perceived as “not good to buy for children” compared to a cereal without it. This effect was smaller among parents, potentially due to children influences of parental decisions during food purchasing.

## Background

Mexico has one of the highest prevalence of childhood obesity worldwide [1–3]**.** Consumption of discretionary foods, like breakfast cereals that contain high amounts of saturated fat and/or added sugar, is a major contributor to childhood obesity [4–6]. These discretionary foods represent 25% of daily caloric intake among Mexican school-aged children and adolescents [7]. Most breakfast cereals available in the Mexican market (69%) in 2014 were classified as “less healthy” according the United Kingdom Nutrient Profiling Model [8]. Furthermore, in Mexico, ready-to-eat breakfast cereals provide 7% of the total energy intake among Mexican preschoolers [9].

The food industry often uses cartoon characters on their product packaging to heighten appeal among children. Socio-cognitive theories and interaction models suggest that children tend to have positive associations with familiar and likable characters. The positive feelings associated with these characters will transfer to the product or brand, increasing brand preference, loyalty, and recognition [10, 11]. Indeed, it has been widely documented that in children and youth, cartoon characters on product packaging attract more attention, [12, 13] increase products’ appeal, [5] and even change perceptions of product taste [6].

A review of literature of eleven studies published between 2004 and 2014 conducted mainly in the USA and European countries reported that cartoon characters may increase children’s appetite, preference for, choice and intake of foods compared with no character branding, especially for energy dense and nutrient-poor foods (e.g. cookies, candy or chocolate) [11]. Studies suggest that cartoon characters help children recognize the brand, [14] and aim to create a positive attitude and loyalty towards the product [15–17]. Recently, the European Consumer Organization stated a position which calls for food manufacturers to stop marketing strategies like the use of brand mascots and licensed media characters marketed to children [18].

However, the effect of cartoon characters on the front of the pack of processed foods among adults is scarce. A study among Canadian parents reported that although they considered products with a cartoon character as more appealing than those without them, they perceived products with a cartoon character as of lower nutritional quality when compared to products with a front-of-pack nutritional label or a cartoon character plus a front-of-pack nutritional label [19]. Parents may be persuaded to buy food products by two factors: 1) perceived product healthfulness inferred from the nutrition information on the package, and 2) their children preferences - which are strongly influenced by cartoon characters - even when the food content may not be nutritious at all [19–21]. Nonetheless, to our knowledge, no study has explored the effect of cartoon characters among non-parents.

Further, studies have reported that the use of nutritional information on the front-of-the pack, such as the front-of-pack nutrient labeling, is higher among females, older adults, those with higher income and education levels, those with a health condition or being the primary shopper of the household [22–24] Based on this evidence, it could be hypothesized that a cartoon character displayed on the front-of-the pack could have a differential effect across groups of people with different individual characteristics. Therefore, the aim of this study was to explore the impact of a cartoon character on Mexican adult consumers’ belief that breakfast cereals are good to buy for children and if individual characteristics, such as having children, modified this effect.

## Material and methods

We analyzed data from the Mexico administration of the first wave of the International Food Policy Study, a cross-sectional survey of adults aged 18–65 years (*n* = 19,857) from five countries, including Mexico, who completed an online questionnaire in December 2017. The survey assessed seven primary policy domains: price/taxation, food packaging and labeling, retail food policies, food marketing (including the current experiment), nutritional labeling in restaurants, nutrition information and education, and food guide/dietary recommendations.

The study sample was recruited via Nielsen Consumer Insights Global Panel and their partners’ panels. The panels were originally recruited using both probability and non-probability sampling methods in each country. Nielsen drew stratified random samples from the online panels in each country, based on known proportions in each age group. Individuals were eligible to participate if they were 18–64 years of age and resided in the target country.

In Mexico, a total of 68,336 email invitations (with a unique link) were sent to a random sample of panelists (after targeting for age). Participation rate was 6.2% (*n* = 4268). All potential respondents were provided with information about the study and were asked for an informed consent prior to completing an online survey.

Most survey items were adapted from national surveys and prior studies. Native Spanish-speakers verified the accuracy of the translation of survey items from English to Spanish. The mean survey time was 42 min. Respondents received compensation in accordance with their panel’s usual incentive structure (e.g., points-based or monetary rewards, chances to win prizes). The study was reviewed by and received ethics clearance through a University of Waterloo Research Ethics Committee (ORE# 21460). A full description of the study methods can be found in the International Food Policy Study: Technical Report – Wave 1 (2017) at http://www.foodpolicystudy.com/methods.

### Cartoon character experiment

Researchers selected an image of a cartoon character commonly used in products marketed to children in Mexico. The Minion character, a computer animated character, became popular from the movies released in Mexico in the years of 2010, 2013, 2015, and 2017.

**Fig. 1:**
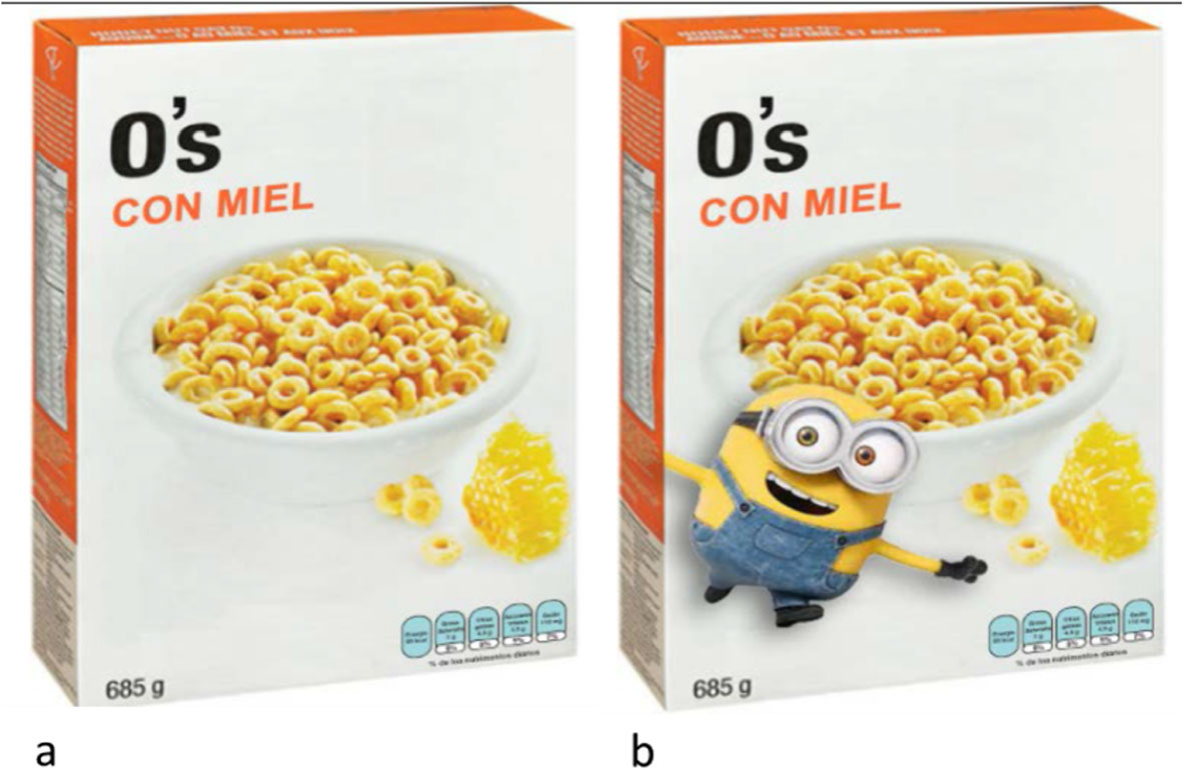
Breakfast cereal shown in the experiment. **a**) Control **b**) Cartoon

Participants were randomly assigned to the “Cartoon condition”, that was a ready-to-eat breakfast cereal box with the Minion character on the front of the package, or the “Control” condition that was the same cereal box with no character on the front of the package **(**Fig. 1**)**. The cereal box displayed a fabricated brand to control for established beliefs about cereal brands. Guideline Daily Allowances, the mandatory front-of-pack label in Mexico, were displayed on the boxes. However, the label was intentionally small and blurred to prevent consumers from reading it. The assigned image was displayed on screen and participants were asked: *“Is this a good cereal to buy children?”* with the response options “Yes”, “No” or “Don’t know”.

### Sociodemographic characteristics

Sociodemographic characteristics included age (categorical), gender (male/female), ethnicity (minority/majority), parental status (no children or having children), education level (high school or lower/technical school/university degree or higher), and region of the country (North/South/Centre/Mexico City). Income was assessed with the question “Thinking about your total monthly income, how difficult or easy is it for you to make ends meet?”, with responses collapsed into “very difficult or difficult”, “neither easy nor difficult”, and “easy or very easy”.

### Other measures relevant to food choices

Self-reported nutrition knowledge was assessed with the question “How would you rate your nutrition knowledge?”, with responses collapsed into not knowledgeable (“not at all knowledgeable” and “a little knowledgeable”), somewhat knowledgeable, and knowledgeable (“very knowledgeable” and “extremely knowledgeable”).

Front-of-pack label understanding was assessed with the question “how difficult or easy is it to identify unhealthy foods using food labels?”, with answer options collapsed into “Very difficult or difficult”, “Neither difficult or easy” and “Easy or very easy”. Front-of-pack label use was assessed with the question “how often do you use nutrition information on food labels when deciding to buy a food product?” with responses recoded to “never or rarely”, “sometimes” and “most of the time or always”.

Label influence in food choice was assessed with the question “Overall, how much do food labels influence what you eat?”, with answer options collapsed into “no influence or a little influence”, and “some or a lot of influence”.

Daily calorie counting was assessed by asking “Do you count calories you consume each day?” and answer options were collapsed into “never or rarely” and “sometimes or most of the time”.

### Data analysis

A total of 4057 adults completed the survey. For the primary outcome, participants were classified as considering the cereal as “Good to buy for children” (answered “Yes”), “Not good to buy for children” (answered “No”) and “Don’t know” (answered “Don’t know”). We removed from the analysis participants with missing data in the outcome (*n* = 19), demographics (e.g., parental status, income adequacy) (*n* = 110) or other measures relevant to food choices (e.g., nutrition knowledge, label use, counting calories) (*n* = 173). This decision was made based on preliminary results showing that randomization was not successful in creating comparable groups. Thus, all models were adjusted for demographic variables and other measures related to food choices. The proportion of participants removed did not differ across experimental conditions (Control group; *n* = 148 and Cartoon group: 154, *p* = 0.252). A total sample of 3755 (Control: 1966; Cartoon: 1789) participants were included in the analyses.

We compared demographic characteristics and information relevant to food choices by experimental condition using t-tests for continuous variables and Chi-square tests for categorical variables. We estimated multinomial logistic regression models to estimate the relative risk ratios (RRR) for considering the breakfast cereal as “Good to buy for children”, “Not good to buy for children” or responding “Don’t know” across experimental conditions (cartoon character or control). Models were adjusted for age, gender, ethnicity, parental status, education level, region of the country, income adequacy, self-reported nutrition knowledge, front-of-pack label understanding, use, and influence on purchasing decisions and daily calorie counting.

Based on previous literature showing differences in the use of nutritional information on the front-of-the pack of processed foods [22–24], we aimed to explore if the effect of the cartoon character differed across specific groups of participants (i.e. gender, age group, health status, education level, parental status, income adequacy, nutritional knowledge and front-of-pack label understanding, use and influence on food purchasing decisions). Separate regression models were used to estimate differences in the associations between socio-demographic correlates and the outcome. Multiplicative interactions between individual characteristics and experimental condition (e.g. experiment group x gender) were entered individually into the base model and interactions that were significant at a level of *p* < 0.05 were then entered into the base model simultaneously. Interactions that were not significant at *p* < 0.05 from the multivariable model were removed, leaving only significant interactions in the final interaction model. Stratified models are presented if the interaction term was significant at a level of *p* < 0.05. Analysis were conducted using Stata SE v14.

## Results

Table 1 shows the characteristics of participants by experimental condition. Experimental groups were comparable in most of their characteristics, except for income adequacy in the subgroup “Difficult” (Control: 38.6% vs Cartoon: 40%) and “neither difficult nor easy” (Control: 41.4% vs cartoon: 37.4%, *p* = 0.029), and for label understanding in the subgroups “neither difficult nor easy” (control: 35.1% vs cartoon 31.1%) and “easy or very easy” (control: 34.8% vs cartoon: 38.8%).

**Table 1:** Comparison of sociodemographic characteristics and other measures relevant to food choices among the overall sample and across parental status condition (*n* = 3755)

		Control box	Cartoon Character box	*p* value
		1966	1789	
	n	% (95% CI)	% (95% CI)	
	3755	52.4 (50.8, 54)	47.7 (46.1, 49.3)	
**Age**				
years	3755	33.8 (0.26)^a^	34.0 (0.27)^a^	0.641
**Age group**				
18–24 y	932	25.6 (23.8, 27.6)	23.9 (22, 26)	0.406
25–30 y	859	22.2 (20.4, 24.1)	23.6 (21.7, 25.6)	
31–39 y	862	22.8 (21, 24.7)	23.1 (21.2, 25.1)	
40–49 y	661	17.8 (16.1, 19.5)	17.4 (15.7, 19.3)	
50–59 y	343	8.6 (7.4, 9.9)	9.7 (8.4, 11.2)	
60–64 y	98	3.0 (2.3, 3.8)	2.2 (1.6, 3)	
**Gender**				
Male	1876	49.8 (47.6, 52)	50.1 (47.8, 52.5)	0.834
Female	1879	50.2 (48, 52.4)	49.9 (47.5, 52.2)	
**Ethnicity**				
Not indigenous	3282	88.1 (86.7, 89.5)	86.7 (85.2, 88.3)	0.213
Indigenous	473	12.0 (10.6, 13.4)	13.3 (11.8, 14.9)	
**Parental status**				
No children	1890	50.9 (48.7, 53.1)	49.7 (47.5, 52.1)	0.494
Having children	1865	49.1 (47, 51.4)	50.3 (48, 52.6)	
**Education level**				
High school or lower	669	18.0 (16.3, 19.6)	17.7 (16, 19.5)	0.201
Technical school	468	11.5 (10.2, 13)	13.5 (11.9, 15.1)	
University degree or higher	2618	70.5 (68.6, 72.6)	68.8 (66.7, 71)	
**Region of the country**				
North	886	23.4 (21.6, 25.4)	23.8 (21.8, 25.8)	0.566
Center	1216	32.6 (30.6, 34.7)	32.1 (30, 34.3)	
Mexico City	884	22.8 (21, 24.7)	24.4 (22.4, 26.4)	
South	769	21.2 (19.4, 23)	19.7 (18, 21.6)	
**Income adequacy (Making ends meet)**				
Difficult	1475	**38.6 (36.5, 40.8)**	**40.0 (37.9, 42.4)**	**0.029**
Neither difficult nor easy	1483	**41.4 (39.3, 43.6)**	**37.4 (35.2, 39.7)**	
Easy	797	**20.0 (18.3, 21.9)**	**22.6 (20.6, 24.5)**	
**Nutritional knowledge**				
A little	1165	31.0 (29, 33.1)	31.0 (29, 33.3)	0.994
Somewhat	2052	54.8 (52.6, 57)	54.6 (52.3, 56.9)	
Very knowledgeable	538	14.2 (12.8, 15.9)	14.4 (12.8, 16)	
**Front-of-pack label understanding**				
Easy or nor difficult	1378	**34.8 (32.7, 36.9)**	**38.8 (36.6, 41.1)**	**0.013**
Neither difficult nor easy	1247	**35.1 (33.0, 37.3)**	**31.1 (28.9, 33.3)**	
Difficult or very difficult	1130	**30.0 (28.1, 32.1)**	**30.1 (28.1, 32.1)**	
**Front-of-pack label use**				
Never or rarely	1634	42.9 (40.7, 45.1)	44.2 (42, 46.6)	
Sometimes	1352	37.3 (35.3, 39.6)	34.5 (32.3, 36.7)	0.159
Most of the times/always	769	19.8 (18, 21.5)	21.3 (19.4, 23.2)	
**Front-of-pack label influence on purchasing decisions**				
No or a little influence	1111	29.2 (27.3, 31.3)	30.0 (27.9, 32.1)	0.623
Some or a lot of influence	2644	70.8 (68.8, 72.8)	70.0 (68, 72.2)	
**Counting calories**				
Sometimes/Most of the time	1117	29.2 (27.3, 31.3)	30.3 (28.2, 32.5)	0.482
Never or rarely	2638	70.8 (68.8, 72.8)	69.7 (67.6, 71.9)	

Differences were tested using Chi-square tests or t-student test for age in years. CI=Confidence Interval

^a^Mean (S.D.)

The mean age was 33.8 years (SD = 0.26) in the Control group and 34.0 years (SD = 0.27) in the Cartoon group. Participants were evenly distributed among males and females; and between those having and those not having children. Most participants were non-indigenous (87.4%), 49.7% were parents, nearly 70% had a university degree or higher, 32.4% lived in the Center of the country, 54.7% reported to be somewhat nutrition knowledgeable, around 70% considered labels had some or a lot of influence in food choices, and nearly 70% never counted calories.

Table 2 shows the proportions and relative risk ratios for considering the breakfast cereal “good to buy for children”, “not good to buy for children” or responding “don’t know” across experimental conditions. A total of 54.8% of participants considered the breakfast cereal as “Good to buy for children” when the box did not display a cartoon character; meanwhile, this figure was of 45.5% when the box displayed a cartoon character. In contrast, 28.9% of participants considered the breakfast cereal as “Not good to buy for children” in the control group, whereas this proportion was of 39.4% in the cartoon character group. The proportion of participants answering “Don’t know” was similar across experimental groups (Control box: 16.3%, Minion box: 15.1%).

**Table 2:** Proportions and adjusted^1^ relative risk ratios (RRR) for considering the breakfast cereal as “Not good to buy for children” or answering “Don’t know” among the overall sample and across parental status condition (*n* = 3755)

	Control Box		Cartoon Character Box	
	n (%)	RRR	n (%)	RRR (95%CI)
**Overall sample** (*n* = 3755)	1966 (100)		1789 (100)	
Good	1077 (54.8**)**	1.00	814 (45.5**)**	1.00
Not good	569 (28.9)	1.00	704 (39.4)	**1.67 (1.45, 1.94)**
Don’t know	320 (16.3**)**	1.00	271 (15.1)	1.15 (0.95, 1.39)
**Parents** (*n* = 1865)	966 (100)		899 (100)	
Good	543 (56.2)	1.00	450 (50.1)	1.00
Not good	272 (28.2)	1.00	314 (34.9)	**1.39 (1.13, 1.72)**
Don’t know	151 (16.6)	1.00	135 (15.0)	1.08 (0.83, 1.42)
**Non-parents** (*n* = 1890)	1000 (100)		890 (100)	
Good	534 (53.4)	1.00	364 (40.9)	1.00
Not good	297 (29.7)	1.00	390 (43.8)	**2.01 (1.63, 2.47)**
Don’t know	169 (16.9)	1.00	136 (15.3)	1.25 (0.95, 1.64)

^1^RRR’s were estimated using multinomial regression models adjusted for age, gender, ethnicity, parental status, education level, region of the country, income adequacy, self-reported nutrition knowledge, front-of-pack label understanding, use, and influence on purchasing decisions and daily calorie counting

**Bolds** indicate significant (*p* < 0.05) associations

The adjusted model showed that participants who viewed a cereal box with a cartoon character were 1.67 (1.45–1.94) times *more* likely to consider the cereal as being “Not good to buy for children” relative to those who viewed a control cereal box (Table 2).

The interaction model showed that this effect was different across parental status categories (Interaction term RRR: 1.39, *p* = 0.029) (Supplementary Table 1). The stratified model across parental status showed that non-parents assigned to the cartoon condition were 2.01 (1.63, 2.47) times more likely to consider the cereal box as “Not good to buy for children” compared to those assigned to the Control condition, whereas this effect was significantly smaller among parents (RRR = 1.39, (1.13, 1.72]) (Table 2). No differences were observed in the proportion of participants answering Don’t know across experimental groups in any of the models.

## Discussion

In this experiment, we tested the impact of a cartoon character on Mexican adult consumers’ belief that breakfast cereals are good to buy for children and if individual characteristics modified this effect. We found that Mexican adults who viewed a cereal box with a cartoon character were *more* likely to consider the cereal as “not good to buy for children” compared to those who viewed the same cereal box without the cartoon character. This effect was stronger among non-parents compared to parents.

Cartoon characters are effective in influencing children’s food preferences, choices and intake, especially for energy-dense and nutrient-poor foods compared with fruits or vegetables [11]. However, few studies have explored the effect of cartoon cereals among adult populations. A study among Canadian parents reported that products with a cartoon character were perceived as of lower nutritional quality when compared to products without these characters [19]. In line with these results, our study showed that cartoon cereals increased the odds of considering the cereal as “not good to buy for children”. Interestingly, this effect was smaller among parents. Differences in the effect between parents and non-parents might be due to the subjective understanding of “good”. It is probable that among parents this word was interpreted as “accepted”, “appealing” or “tasty” for their children, instead of “healthy” for children. However, we believe this explanation is rather unlikely given that in Spanish the word “good” describing a food is generally used for “positive value”, “high quality”, “healthy” “nutritious” or “that its consumption does not cause adverse effects” [25].

Also, children have been identified as the major influencers within the family decision making unit [26, 27]. Therefore, other possible explanation to the differences in the effect of cartoon characters between parents and non-parents might be due to actual influences of children preferences on parental decisions but evidence is not consistent among populations. In the UK, 34% of sales of food are driven by children nagging [28] and 40–80% of children requests of foods were granted [29]. A study in Scottish parents, showed that they may grant children requests of foods, despite knowing their child’s demand was for junk food [30]. Conversely, a study in the UK found that parents claimed not to give in to their children’s requests to purchase unhealthy foods [31]. Similarly, in an experiment carried out in Australia, parents were not affected in their food choices by the presence of a cartoon character, regardless of whether the character may have appealed to children or represented a sporting activity, but they counted with the advantage of the Health Star Rating front-of-pack label that was the main contributor of the food choices [32]

In our study, Guideline Daily Allowances, the mandatory front-of-pack label in Mexico, were displayed on the boxes. However, the label was intentionally small to prevent consumers from reading it, thus the label was unlikely to affect the answers. Future studies could explore this relation to clarify the contribution of children preferences to their parents’ food selection, considering the effect of cartoon characters displayed in food products directed to children and their effect while front-of-pack labels are displayed along with marketing strategies [33]. Finally, the effect size of the cartoon character found in our study may be considered small. In our study, 29% of participants in the control group and 39% in the cartoon character group considered the breakfast cereal as “not good to buy for children”, a difference of 10.4 percentage points between groups. This effect was stronger among non-parents (14 percentage points).

To our knowledge, no other study among parents or adults has explored the proportion of participants considering a food product as “good” or “healthy” when a cartoon character is displayed on the front of the pack. Similarly, studies conducted among children have rarely explored the effect of cartoon characters on diet quality perceptions [11]. However, studies among children exploring other outcomes (e.g. food choices) have usually reported larger differences between groups. For example a study among Guatemalan children (4–9 years of age) found that cartoon characters increased the taste preference and snack choices between 20 to 40 percentage points [34]. Taken together, results suggest that while cartoon characters may promote the consumption of specific foods among children [11], an opposite or null effect on healthiness perception is observed among adults and parents.

To our knowledge, this is the first nation-wide study in Latin America evaluating the impact of cartoon characters on perceptions of processed foods. However, our study has limitations that should be acknowledged. First, the sample consisted mostly of participants of high socioeconomic and educational levels with access to internet, as is commonly observed when using internet-based data collection approaches. According to the 2015 National Inter-Census Survey, 18.6% of Mexican population older than 15 years have a university degree or more [35], which is much lower than the ≈70% of participants with this education level in our sample. Considering that in Mexico 50.9% of households have access to internet [36] and that the average education level is considerably lower to the one reported in this study, the external validity of our findings is limited to those with similar characteristics to the ones of participants. Thus, our findings mostly reflect the effect of cartoon characters on the perceived goodness of breakfast cereals among high income Mexicans.

However, we believe that our results provide important insights for understanding the impact of cartoon characters among high SES groups in Mexico, which until now has been understudied. Another limitation of this study was that we did not assess the impact of a broader range of cartoon characters on adults’ perceptions. Cartoon characters are hypothesized to influence food preferences through their familiarity and likability particularly among children. However, this study was unable to recreate the real-world experience of shopping for cereal while considering children’s preferences, which influence parental food purchasing behavior [11, 30].

## Conclusion

Among this sample of Mexican adults, a cereal with a cartoon character on the packaging was more often perceived as “not good to buy for children” compared to a cereal without the cartoon. This effect was smaller among parents, probably due to children influences of parental decisions during food purchasing. Future studies should continue exploring the effect of this and other marketing strategies used by the food industry among a more diverse population to identify potential strategies that help consumers make informed and healthy choices.

## Supplementary information

**Additional file 1: Table S1.** Multinomial regression model with interaction between parental status and experimental group (*n* = 3755)

## Data Availability

The datasets used and/or analyzed during the current study are available from the corresponding author on reasonable request.
