# Supplementary material for: The impact of a cartoon character on adults perceptions of Children’s breakfast cereals: a randomized experiment
Source: Nutr J. 2020 May 17;19:43. doi: 10.1186/s12937-020-00565-5 (PMC7232833; doi:10.1186/s12937-020-00565-5)
Supplement: Supplementary file 1 — Additional file 1: Table S1. Multinomial regression model with interaction between parental status and experimental group (n = 3755) [file 12937_2020_565_MOESM1_ESM.docx]

**Supplementary Table 1.** Multinomial regression model with interaction between parental status and experimental group (n=3,755)

|  |  |  | **"Good"** |  | **"Not good"** | |  | **“Don't know”** | |
| --- | --- | --- | --- | --- | --- | --- | --- | --- | --- |
| **“No good” (ref. “Good”)** | **Subcategories** |  | **RRR (95% CI)** |  | **RRR (95% CI)** | **p value** |  | **RRR (95% CI)** | **p value** |
| **Experimental group** | Control box |  |  |  | 1.00 | 0.001 |  |  | 0.542 |
|  | Cartoon character box |  | 1.00 |  | **1.41 (1.14, 1.74)** |  |  | 1.09 (0.84, 1.43) |  |
| **Parental status** | Parents |  |  |  | 1.00 | 0.031 |  |  | 0.092 |
|  | Non-parents |  | 1.00 |  | **1.27 (1.03, 1.58)** |  |  | 1.26 (0.97, 1.65) |  |
| **Interaction term**  **Experimental group and Parental status** | Cartoon character  and Non-parents |  | 1.00 |  | **1.39 (1.04, 1.85)** | 0.029 |  | 1.14 (0.78, 1.66) | 0.527 |

^1^ Model adjusted for age, gender, ethnicity, parental status, education level, region of the country, income adequacy, self-reported nutrition knowledge, front-of-pack label understanding, use, and influence on purchasing decisions and daily calorie counting.

**Bolds** indicate significant (p<0.05) associations
